# Indonesian infertility patients’ health seeking behaviour and patterns of access to biomedical infertility care: an interviewer administered survey conducted in three clinics

**DOI:** 10.1186/1742-4755-9-24

**Published:** 2012-09-28

**Authors:** Linda Rae Bennett, Budi Wiweko, Aucky Hinting, IB Putra Adnyana, Mulyoto Pangestu

**Affiliations:** 1 Nossal Institute for Global Health, The University of Melbourne, Parkville 3010, Australia; 2 Department of Obstetrics and Gynaecology, School of Medicine, The University of Indonesia, Jakarta 10430, Indonesia; 3 Department of Obstetrics and Gynaecology, School of Medicine, Airlangga University, Surabaya 60115, Indonesia; 4 Department of Obstetrics and Gynaecology, School of Medicine, Udayana University, Bali 80232, Indonesia; 5 Education Program in Reproductive Biology, Department of Obstetrics and Gynaecology, Monash University, Clayton 3168, Australia

**Keywords:** Indonesia, Infertility care, ART, Access, Equity, Female infertility, Reproductive rights, Patient education, Referral systems, Cost of health care

## Abstract

**Background:**

Indonesia has high levels of biological need for infertility treatment, great sociological and psychological demand for children, and yet existing infertility services are underutilized. Access to adequate comprehensive reproductive health services, including infertility care, is a basic reproductive right regardless of the economic circumstances in which individuals are born into. Thus, identifying and implementing strategies to improve access to assisted reproductive technology (ART) in Indonesia is imperative. The principle objectives of this article are to improve our understanding of infertility patients’ patterns of health seeking behaviour and their patterns of access to infertility treatment in Indonesia, in order to highlight the possibilities for improving access.

**Methods:**

An interviewer-administered survey was conducted with 212 female infertility patients recruited through three Indonesian infertility clinics between July and September 2011. Participants were self-selected and data was subject to descriptive statistical analysis.

**Results:**

Patients identified a number of barriers to access, including: low confidence in infertility treatment and high rates of switching between providers due to perceived treatment failure; the number and location of clinics; the lack of a well established referral system; the cost of treatment; and patients also experienced fear of receiving a diagnosis of sterility, of vaginal examinations and of embarrassment. Women’s age of marriage and the timing of their initial presentation to gynaecologists were not found to be barriers to timely access to infertility care.

**Conclusions:**

The findings based on the responses of 212 female infertility patients indicated four key areas of opportunity for improving access to infertility care. Firstly, greater patient education about the nature and progression of infertility care was required among this group of women. Secondly, increased resources in terms of the number and distribution of infertility clinics would reduce the substantial travel required to access infertility care. Thirdly, improvements in the financial accessibility of infertility care would have promoted ease of access to care in this sample. Finally, the expansion of poorly developed referral systems would also have enhanced the efficiency with which this group of patients were able to access appropriate care.

## Background

The desirability and feasibility of providing infertility treatment in developing country settings has been widely debated [1-4]. Our position in this article is that access to infertility care is a basic human right that is equally applicable to all people regardless of the economic circumstances they are born into. This position reflects not only a strong commitment to the principles of equity in health care and human rights, but also acknowledges the desires of everyday Indonesians who will continue to struggle to overcome infertility regardless of the theoretical debates circulating in realms vastly removed from the realities of their lives.

Indonesia currently has 20 government regulated fertility clinics with state of the art technology. These clinics offer a wide range of diagnostic procedures, as well as IVF and intracytoplasmic sperm injection (ICSI) to married Indonesian couples^a^. However, utilization of these services remains very low when compared to the number of IVF cycles performed elsewhere in the region. Indonesia’s rate of IVF uptake is also startlingly low relative to the size of its population. For instance, Singapore with an estimated population of 5 million residents recorded 2500 IVF cycles in 2010, while Indonesia with an estimated population of 237 million performed 2627 cycles in the same year. The highest number of IVF cycles in the region for 2010 was in Vietnam where 6000 cycles were performed^b^.

Low rates of IVF uptake and restricted access to biomedical care in Indonesia cannot be explained simply as a function of poor capacity. ART clinics are heavily clustered in large cities on the island of Java and the distribution of patients and IVF cycles is uneven between existing clinics. While some clinics are working close to their capacity - performing between 300 and 400 cycles a year, others are performing less than 100 cycles annually and report working at around 30% of their capacity. Thus, the infertility care system as a whole is experiencing an oversupply in available services^b^. Expansion of the industry is occurring rapidly, there were 16 operating clinics at the time of data collection in 2011and 4 more have subsequently opened in 2012.

The Indonesian situation is further complicated by a combination of excess service capacity and high levels of infertility among the population. Estimates of infertility rates vary, with the most conservative rates being between 10% and 15% of the reproductive aged population^c^. These rates are extrapolated based on the numbers of patients seeking biomedical fertility care and are likely to be underestimates as they fail to include those people who do not seek biomedical solutions to their fertility concerns. Alternatively, based on data collected in Indonesia’s Demographic Health Survey - 2002, the World Health Organization (WHO) has estimated the female infertility rate (combining primary and secondary infertility) to be 22.3% of married women between the ages of 15 and 45 [5].

Regardless of the difficulties in establishing accurate infertility rates for couples, we can be confident that infertility is a major concern in Indonesia both in terms of the real numbers of people affected and the cultural significance of childlessness. The desire for children is virtually universal among married Indonesian couples and the cultural definition of a family is dependent upon having children [6]. Based on the current age structure of the population there are approximately 39.8 million Indonesian women of childbearing age. Even when applying the conservative estimate of a 10% infertility rate this translates to a sub-population of around four million women experiencing infertility. Employing the accepted wisdom that approximately 5% of infertile couples will be eligible candidates for IVF we can infer that Indonesia has a demand for 200,000 cycles upwards. When we apply realistic economic logic to these numbers, accepting that only 10% of infertile Indonesian couples are in an income band where the cost of ART would be reasonably affordable, the result is still 20,000 eligible couples^d^. Clinic attendance records for 2011 from across Indonesia indicated that approximately 22,000 couples sought biomedical infertility treatment for the year. This population of 22,000 infertile couples who were actively seeking biomedical infertility treatment in 2011 was our target population. Thus, our final sample size of 212 female patients represents approximately 1% of the target population for the study.

The principle objective of this article is to improve our understanding of current patterns of health seeking behaviour and access to infertility care among existing infertility patients in Indonesia. The second objective is to highlight the possibilities for improving patients’ access to infertility care. A secondary goal of the article is to expand the currently sparse body of published research on infertility care in developing country contexts. Access to infertility care can be understood as a function of many interrelated components, in this article we focus on understanding access to care via an analysis of data collected from female patients.

## Methods

While clinical research and retrospective studies of ART outcomes are well established in Indonesia social research into infertility and its treatment is still an emerging field. The data presented in this article was generated by the “Survey of Indonesian infertility patients’ reproductive knowledge and health seeking behaviour” conducted between July and September 2011. This exploratory study was innovative in the Indonesian context because of its explicit focus on patient perspectives on infertility treatment, which have been absent in prior research. The survey was developed and administered in the national language - Bahasa Indonesia. Formulating and refining survey questions was a collaborative process involving fertility consultants, survey interviewers and volunteers who took part in piloting the survey. The final tool included 110 variables arranged in five sections, which were: 1) socio-demographic data of female participants and their current husbands; 2) female patients’ sources of knowledge about human reproduction and infertility; 3) female patients’ actual knowledge of reproduction and infertility; 4) female patients’ and their husbands’ experiences of biomedical infertility treatment; and 5) patients’ perceptions of their needs in relation to infertility care. This article focuses on data collected in sections 1 and 4 of the survey.

All respondents were infertile women recruited through invitations posted at three infertility clinics in the cities of Jakarta, Surabaya and Denpasar. The sample is therefore self-selected and not randomly selected. The criteria for participation were that women be married and aged between 18 and 45 years, had experienced infertility problems and were currently seeking biomedical treatment, and finally that women were not undergoing IVF programs at the time of the survey. The decision to exclude women undergoing IVF was to ensure that their treatment could not potentially be impacted upon by an increase in stress hormones related to recounting their experiences or feelings about infertility during survey interviews.

Surveys were completed by a total of 232 participants and data was coded by hand and then entered into STATA. A preliminary coding frame was developed prior to data collection and later revised to reflect the actual range of responses received. After cleaning the data 20 surveys were eliminated leaving a final sample size of 212 for analysis. Descriptive statistical analysis was then performed by two statisticians, to allow cross-checking for accuracy. No bivariate or multivariate methods were used in the analysis produced for this paper.

The decision to focus this initial study of Indonesian infertility patients on women was both pragmatic and political. Firstly, women seek out infertility treatment at higher rates than men in Indonesia and are thus more easily accessible as research participants^f^. Secondly, in previous studies with fertility patients in non-Western settings women have been found to be more willing and forthcoming research participants than men [7]. Thirdly, the psychological distress and social suffering reported by Indonesian women experiencing fertility difficulties is greater than that reported by Indonesian men, thus an initial focus on women acknowledges this asymmetrical impact of infertility on them^g,h^. However, this study did collect some data regarding men’s participation in infertility treatment, as reported by their wives.

The choice of which infertility clinics to recruit through was based on the available funding, which was adequate for only three sites, and the desire to include clinics that service clients from a range of socioeconomic groups and geographical areas. The clinics chosen to be involved in the study are situated in different cities and different provinces. The Jakarta based clinic is situated in an elite private hospital and tends to attract clients with a very high income. In Surabaya the chosen clinic is situated within a university teaching hospital and tends to attract both mid to lower income clients. The Denpasar based clinic is situated within a regional public hospital that primarily services clients who cannot afford to pay for private services. Recruitment of respondents was relatively even across three sites - 35% from Jakarta, 36% from Surabaya, and 29% recruited in Denpasar.

When we analyzed the survey sample we found it to be highly indicative of the sub-population of Indonesians who have the easiest access to infertility care due to their affluence, proximity to services and higher levels of education. The sample was comprised of 78% urban residents, with the remaining 22% living in rural areas or poor urban fringe communities on the outskirts of large cities. The ages of women respondents ranged between 18 and 45 years and the median age was 31 years. All women in the sample were literate and 86% had completed senior high school or some form of tertiary education, and 60% possessed a tertiary degree of some kind. Thus, the formal educational attainment of women in the sample was very high, considerably higher than among the broader population^i^.

Income levels among the sample were not reflective of national patterns of income distribution. Indonesia’s national median monthly income is reported to be US$260 and 29% of the population is still classified as living under the poverty line - earning less than US$28 per month [8]. Monthly household incomes among our respondents were more skewed towards higher socio-economic groups with 50% of the sample being classified as middle class or elite on the basis of their monthly house hold income alone. In a nutshell, our sample was well educated, affluent and predominantly urban. This confirmed our expectations that women with lower incomes, less education and those living in more remote areas would be less likely to access infertility clinics, and subsequently would be less likely to be recruited for the survey.

Research participants were supplied with plain language information sheets about the research and asked to provide voluntary written informed consent before interviews were conducted. All respondents were made aware of their rights to skip any questions they did not wish to answer, and to withdraw from participation before survey interviews were concluded and their de-identified data was stored. The interviews were conducted in private counseling rooms and took between 30 and 45 minutes. Anonymity was protected by ensuring that participants’ names were not recorded on survey forms and by storing consent forms separately from completed surveys. The La Trobe University Human Ethics Committee granted principle ethics approval in Australia in May 2011 (HEC 11–011, approved 24 May 2011)^j^. A team of 14 female interviewers who were all doctors, and who received intensive training in research ethics and interviewing techniques, conducted the survey interviews. None of the interviewers were treating doctors of survey respondents. The choice to have interviewers and interviewees of the same gender enhanced rapport and the ease with which respondents discussed their reproductive health. Following each interview respondents were given an information booklet to take home that contained the correct answers to all of the knowledge questions in the survey, and an overview of the prevalence, causes and treatment of infertility in lay language.

Below we present our findings on a range of factors relevant to accessing infertility care, including: 1) the timing of initial presentation to obstetrician/gynaecologists (OBSGYN); 2) the types of providers visited by fertility patients and patterns of referral; 3) the number of OBSGYN visited by patients and their rationale for seeing multiple providers; 4) men’s participation in infertility care; 5) barriers to access reported by patients; 6) patterns of travel for infertility care; and 7) cost and access to care.

## Results

### Timing of presentation among infertility patients

In public discussions surrounding the issue of access to infertility care in Indonesia it has been suggested that Indonesian women have begun to marry too late and to wait too long to access the biomedical system [6]. However, our findings refute these assumptions by revealing that the median age of marriage for women in the sample was only 26 years old and that the median age of women at their first visit to an OBSGYN for fertility care was 28 years of age. When we calculated the length of time women waited after marriage before seeking infertility care we found the mean number of months to be 25 and the median number of months to be 12.

### Types of providers and referral patterns

To gain an overview of where our respondents were seeking assistance for infertility we asked them to list the range of biomedical providers they had visited regarding their fertility concerns. The respondents identified four categories of providers they sought assistance from. All respondents (n = 212) had visited an OBSGYN, 27 women (13%) had visited a general practitioner, 17 women (8%) had visited a midwife, and only 6 women (3%) had visited a nurse regarding infertility. The popularity of different providers appears to follow a clear pattern indicating that women in this sample preferred to access providers with higher levels of specialized reproductive skills and knowledge, with OBSGYN being by far the most popular choice of providers for fertility concerns.

While 27 out of 212 respondents reported having visited a general practitioner (GP) for fertility concerns, only 17 respondents out of 212 (or 8%) reported having received a GP referral to an OBSGYN. Conversely, 92% of the 212 women in the sample reported finding OBSGYN themselves without the assistance of GPs. In the final section of our survey we also included opportunities for respondents to clarify what kinds of information related to their fertility concerns would be most useful to them. The issue of referral to appropriate care was a concern for 5% of 212 women, who requested details of other OBSYGN, despite the fact that they were recruited through infertility clinics.

### Number of OBSGYN visited

To better understand women’s patterns of health seeking, we also recorded the number of OBSGYN visited by respondents. Results are depicted in Table 1 below. The mean number of OBSGYN visited by women was 3 and the median number visited was 2. Thus, most patients had seen between 1 and 3 specialists, 20% had seen between 4 to 6 OBSGYN, while the highest number of OBSGYN visited by a respondent was 12. These results indicate that switching between different providers was common and was the experience of 87% of 212 women. We also inquired about respondents’ knowledge of the qualifications of their most recent OBSGYN, asking if they were able to confirm whether they had seen a doctor who was a qualified fertility specialist (known as K-FER in Indonesia). Only 41% of 212 respondents were able to confirm that the doctor they had seen most recently was a qualified infertility consultant. We also asked respondents to provide reasons why they had switched between doctors. The most common reason was perceived treatment failure, reported by 32% of 139 respondents. The full range of reasons provided by women is presented below in Table 2.

**Table 1:** Number of OBSGYN visited

**Number of OBSGYN visitedby each respondent**	**Number of women**
1 to 3	162 (76%)
4	26 (13%)
5	10 (5%)
6	5 (2%)
7 to 12	9 (4%)

Note: (n = 212).

**Table 2:** Reasons for changing doctors

**Reasons for changing doctors among respondents who hadvisited more than one OBSGYN**	**Percentage of women**
Treatment from initial/earlier doctor was not successful	44 (32%)
Wanted a second opinion	25 (18%)
Heard another doctor had a good reputation	18 (13%)
Not compatible with the first/earlier doctor	17 (12%)
Moved residence	8 (6%)
Wanted a more modern doctor	7 (5%)
Felt initial/earlier doctor did not listen to them	7 (5%)
Other	13 (9%)

Note: (n = 139).

### Men’s participation in fertility care

Gender equity in access to services is essential for the recognition of both women’s and men’s right to reproductive health care, and equal participation of men and women is critical to successful ART outcomes. For this reason we asked women to comment on their husbands’ participation in treatment. Rates of male participation were actually higher than expected, with 93% of 212 women reporting that their husbands had ever accompanied them to a consultation. However, rates of attendance throughout infertility treatment were significantly lower, with only 50% of 212 women reporting that their husbands regularly or always accompanied them to consultations. Distance from the clinic was given as a reason for husbands’ non-attendance by 4% of 106 women, and husbands’ disinterest in infertility treatment was given as a response by 2% of 106 women. Finally, 30% of 106 women reported that their husbands were too busy to regularly attend infertility consultations.

### Barriers to accessing care

Although the women in this sample had all visited OBSGYN for fertility concerns, we did not take it for granted that they had done so without having to negotiate potential barriers to access. To explore this issue we asked all respondents if they had ever felt worried or frightened about visiting an OBSGYN. A total of 84 respondents (or 40% of 212) answered yes to this question. Of these 84 women 77 also provided reasons as to why they felt worried or frightened, and the most significant reason by far was fear of a sterile diagnosis. The full range of reasons given is listed below in Table 3.

**Table 3:** Reasons why women were worried or frightened about visiting OBSGYN

**Reason why respondent was worried or frightened about visiting an OBSGYN**	**Percentage of respondents**
Afraid of sterile diagnosis	47 (61%)
Afraid of vaginal examination	11 (14%)
Afraid of feeling shy or ashamed	10 (13%)
Worried about of cost of treatment	4 (5%)
Had, or had heard about, a bad experience with an OBSGYN	3 (4%)
Other reasons	2 (3%)

Note: (n = 77).

### Patterns of travel for fertility care

Due to the uneven geographical distribution of infertility services in Indonesia we also examined patients’ patterns of travel to access care. The results are presented in Table 4 below. Less than half the sample accessed infertility care without having to travel outside their own district, and 54% of patients had to travel substantial distances to access care.

**Table 4:** Distance travelled for infertility care

**Distance travelled to access infertility care**	**Percentage of respondents**
Able to access care within their own district	97 (46%)
Travelled outside their own district to access care	53 (25%)
Travelled outside of their own province to access care	19 (9%)
Travelled outside of their own island to access care	34 (16%)
Travelled to another country to access care	9 (4%)

Note: (n = 212).

### Cost and access to care

The cost of health care is widely understood as a key factor determining access, and equity in access, to reproductive health care [9]. A total of 19% of 212 respondents explicitly stated that they had found it difficult to pay for infertility care, 26% of 212 respondents reported having to save money to finance infertility care, and 4% of 212 women reported that they had borrowed money to assist them in meeting the costs of care. In sum, 40% of the sample of 212 reported that they could not easily pay for infertility care out of their regular income.

## Discussion

This research has yielded important insights into patterns of health seeking and access to infertility care among female infertility patients in Indonesia. Our findings have been significant for disrupting popular myths regarding women’s behaviour in response to infertility, assumptions that have often led to significant social suffering for Indonesian women. Specifically, we found no evidence that women attending these three clinics were marrying too late or choosing long delays before seeking infertility care. As a result we can shift the focus away from blaming individuals and towards examining how infertility care may be improved to promote better outcomes.

In this sample, a high rate of switching between different OBSGYN for infertility treatment was identified as a key concern. Patients’ explanations of why they were moving between multiple providers revealed that women were frequently dropping out of treatment cycles due to their frustration at not falling pregnant, and as a result missing out on the recommended progression of diagnosis and treatment. An appropriate response to this dilemma is to adequately educate patients from the outset of their treatment, informing them about the gradual progression of diagnosis and treatment, to prevent unnecessary dropouts and maximize treatment success.

The finding that 40% of our sample had been worried or frightened about accessing fertility care indicates the need for improved social awareness through community education. Social awareness is a key component of the infertility care system, and in particular education is needed to ameliorate patients’ fears regarding a diagnosis of sterility, their fear of vaginal examination and the embarrassment often associated with reproductive health problems. By acknowledging and discussing potential fears surrounding infertility treatment it is hopeful that the level of anxiety in the community more generally will decrease and that this will translate into greater willingness to access care. A comparable study, conducted with 150 women experiencing infertility in South Africa by Dyer et. al., similarly concluded that improved health education and counseling urgently needs to be integrated into infertility treatment in the developing world [10].

Patterns of health seeking among women in this study revealed a strong preference for highly qualified providers, but low use of GPs for referral to specialist services. The fact that only 8% of our sample accessed infertility care via GP referral indicates enormous potential for improving the current referral system. Lack of appropriate referral from general practitioners and primary health services has also been identified as a factor limiting access to infertility care even in studies situated in developed Western settings such as the United States [11]. Increasing referral through Indonesian GPs also holds the possibility of ensuring that a greater proportion of patients are referred to specialized infertility consultants (K-FER) rather than generalist OBSGYN. If increased GP referral were to result in patients gaining more targeted access to the most appropriate providers, there could also be a reduction in patient dissatisfaction and subsequent switching between providers.

Our results also showed that while the majority of husbands had attended infertility treatment at least once with their wives, the findings still fall seriously short of the ideal of “treating the couple” where both spouses are present throughout the treatment process. These findings parallel those of the study by Dhont et al. in Rwanda, where men were found to be significantly less present than their wives over the duration of infertility care visits [12].

Patients’ patterns of travel for fertility care indicated that less than half of the respondents were able to access care without leaving their district. This finding highlights the need for geographic expansion of fertility services beyond large cities in Java and Bali. To expand the availability of fertility services across Indonesia continued training and certification of fertility consultants, who are willing to practice in regional areas, is imperative.

Our findings regarding the cost of infertility care provide evidence that the financial structure of the existing infertility system needs to be overhauled, as 40% of this sample reported that they could not pay for fertility care out of their regular income. A move towards low-cost IVF in Indonesia is essential, as access for those even from higher socioeconomic groups in our sample was constrained by the current cost structure of the system. Our finings regarding the financial accessibility of biomedical infertility care in Indonesia reflect the general pattern of affordability in other developing countries documented by Collins, who observed that the cost of one IVF cycle in developing country settings is generally in excess of half the average annual income [13].

While explicitly privileging patient perspectives is a key strength of this research, the exclusion of women with fertility concerns who do not access biomedical care is an obvious weakness. Complimentary research that includes Indonesians who have not sought solutions to infertility within the biomedical sphere is thus needed to complete the access picture. Moreover, research that positions men as key informants on their experiences of infertility and infertility treatment is also important, to illuminate why men participate at lower rates than women, and to balance our understanding of how to combat infertility in Indonesia. This was a cross-sectional study, and thus represents the participants’ experiences of infertility treatment at only one point in time. This limitation means that some variables may be biased because of this. For example, the median number of OBSGYN visited could be underestimated as the respondent may have just begun seeking help and may over time seek out more providers. Moreover, we acknowledge that the sample size of the study was not large, but for an exploratory study we feel it was sufficient, and that it has highlighted the utility of further research with larger sample sizes.

## Conclusions

Based on our research findings we believe there are four key areas that should be addressed to improve access to infertility care and ART for Indonesians. Improving the education and counselling for infertility patients, and promoting greater social awareness about infertility and its treatment among the community in general, are critical areas for intervention where action can be commenced in the relative short term. The referral system for infertility care is another sphere where productive changes can be made. Strengthening the links between general practitioners and infertility providers is a process that is realistic in the medium term, as it involves significant collaboration from within and outside of the existing infertility system. Expansion of the geographical coverage of fertility clinics is a third area for action that should be ongoing until services are far more evenly distributed across the nation. Finally, reducing the out-of-pocket costs of infertility care, through an overhaul of the financial structure of the current system is imperative, and will require significant change over the long term. The gradual shift to a low-cost system will require further scientific research into ways of reducing the cost of IVF in Indonesia, as well as research into what models of service provision for ART are most viable in Indonesia’s resource poor communities. Reductions in the out-of-pocket cost of infertility care could also potentially be supported by a government commitment to subsidizing IVF and the inclusion of infertility treatment in medical insurance policies.

Despite remaining gaps in social research into infertility in Indonesia, we are confident that this exploratory study has provided evidence that can begin to inform a pragmatic response to the imperative of expanding access to biomedical infertility treatment for Indonesians. The authors of this article are engaged in ongoing efforts to extend this exploratory study to conduct a much larger comparable survey of fertility patients recruited through each of Indonesia’s 20 registered fertility clinics.

## Endnotes

^a^Access to ART for unmarried people and same-sex couples is not legally sanctioned in Indonesia. While this raises important issues in terms of who is given legal rights to access health care, we do not discuss the regulatory framework of ART in Indonesia here due to space constraints. We also focus on married women because this was our target group for the study. ^b^These estimates were collected by Budi Wiweko in his role as the Head of the Scientific Division of POGI JAYA (Indonesian Obstetrics and Gynaecology Association, Jakarta Branch). ^c^Unpublished industry profile paper. Wiweko B: Presented at the Annual Meeting of the Indonesian Association for In Vitro Fertilization (PERFITRI). Jakarta, 6 July 2009. ^d^Ibid. ^e^Unpublished conference paper. Wiweko B, and Bennett LR: Fertility patients’ patterns of treatment seeking: Understanding delays in presentation and barriers to access. Presented at The 14th World Congress on Human Reproduction, Melbourne, 30 November – 3 December 2011. ^f^Ibid. ^g^Unpublished conference paper. Bennett LR: *Sudah menikah tetapi bukankeluarga*: Infertility and social suffering in Indonesia. Presented at the 8^th^ Annual Conference of the Indonesian Association of Obstetrics and Gynaecology (PIT POGI 18), Jakarta, 7–9 July 2010. ^h^Unpublished conference paper. Dwipayana IM: Different degrees of depression among husbands and wives in couples with infertility problems. Presented at Claiming Sexual and Reproductive Rights in Asian and Pacific Societies – the 6^th^ Asia Pacific Conference on Reproductive and Sexual Health and Rights. Yogyakarta, 19–22 October 2011.^i^The female participation rate in tertiary education in urban areas in Indonesia has only recently reached 15% [14]. ^j^The principle investigator Dr Linda Bennett was located at La Trobe University at the commencement of this project. She later relocated to The University of Melbourne where retrospective ethics approval was granted on the basis of the original application approved by the La Trobe University Human Ethics Committee. The research also received ethical approval from all other universities and hospitals collaborating on the project. The relevant ethics bodies included: The Ethics Committee of the School of Medicine at the University of Indonesia; The Hospital Ethics Committee of Kencana Hospital (Jakarta); The Ethics Committee of the Medical School of Airlangga University; The Hospital Ethics Committee of Siloam Hospital (Surabaya); and The Hospital Ethics Committee of Sanglah District Hospital (Denpasar).

## Abbreviations

ART: Assisted reproductive technology; GP: General practitioner; K-FER: Qualified fertility specialist; IVF: In vitro fertilization; ICSI: Intracytoplasmic sperm injection; OBSGYN: Obstetrician gynaecologist; PERFITRI: Indonesian Association for In Vitro Fertilization; POGI: Indonesian Obstetrics and Gynaecology Association; WHO: World Health Organization.

## Competing interests

The authors declare that they have no competing interests.

## Authors’ contributions

LRB was the principle investigator for this study and BW was the Indonesian co-investigator. MP played an instrumental role in forming the research team. All authors participated in developing the study design and the survey tool. LRB trained all survey interviewers and BW, AH and IBPA supervised data collection in the three field sites. LRB worked with the project statisticians to produce the study findings. LRB and BW wrote the initial draft of the paper and all other authors participated in refining the analysis and approved the manuscript.

## References

[B1] van Balen F Gerrits T Quality of infertility care in poor-resource areas and the introduction of new reproductive technologies Hum Reprod 2001 16 2 215 219 10.1093/humrep/16.2.215 11157809

[B2] Pennings G Ethical issues of infertility treatment in developing countries ESHRE Monograph 2008 15 20 10.1093/humrep/den1442

[B3] Ombelet W False perceptions and common misunderstandings surrounding the subject of infertility in developing countries ESHRE Monograph 2008 8 11 10.1093/humrep/den204

[B4] Makuch MY de Padua KS Petta CA Osis MJD Bahamondes L Inequitable access to assisted reproductive technology for the low-income Brazilian population: a qualitative study Hum Reprod 2011 26 2054 2060 10.1093/humrep/der158 21613314

[B5] Rutstein SO Shah IH Infecundity, Infertility, and Childlessness in Developing Countries. DHS Comparative Report No. 9 2004 Calverton: ORC Macro and the World Health Organization

[B6] Bennett LR Infertility, womanhood and motherhood in contemporary Indonesia: understanding gender discrimination in the realm of biomedical fertility care Intersections: Gender and Sexuality in Asia and the Pacific 2012 28 http://intersections.anu.edu.au/issue28/bennett.htm

[B7] Kohler Riessman C Inhorn MC, Balen F Positioning gender identity in narratives of infertility Infertility Around the Globe: New Thinking on Childlessness, Gender, and Reproductive Technologies 2002 Berkley: University of California Press 152 170

[B8] Central Bureau of Statistics - Indonesia (BPS) Number and Percentage of Poor People, Poverty Line, Poverty Gap Index, Poverty Severity Index by Province 2011 [http://dds.bps.go.id/eng/tab_sub/view.php?tabel=1&daftar=1&id_subyek=23&notab=1]

[B9] Gamble Kelley A Reducing Financial Barriers to Reproductive Health Care: Experiences with Free Care and Health Insurance 2010 Aspen: Ministerial Leadership Initiative for Global Health Issue Brief, Aspen Global Health and Development http://www.ministerial-leadership.org/sites/default/files/events/event_files/MLI%20Issue%20Brief_Reducing%20Financial%20Barriers%20to%20Reproductive%20Health%20Care.pdf

[B10] Dyer SJ Abrahams N Hoffman M van der Spuy ZM Infertility in South Africa: women’s reproductive knowledge and treatment-seeking behaviour for involuntary childlessness Hum Reprod 2002 17 6 1657 1662 10.1093/humrep/17.6.1657 12042294

[B11] Jain T Hornstein MD Disparities in access to infertility services in a state with mandated insurance coverage Fertil Steril 2005 84 1 221 223 10.1016/j.fertnstert.2005.01.118 16009188

[B12] Dhont N Luchters S Ombelet W Vyankandondera J Gasarawabwe A van de Wijgert J Temmerman M Gender differences and factors associated with treatment-seeking behaviour for infertility in Rwanda Hum Reprod 2010 25 2024 2030 10.1093/humrep/deq161 20573675

[B13] Collins J An international survey on the health economics of IVF and ICSI Hum Reprod Update 2002 8 265 277 10.1093/humupd/8.3.265 12078837

[B14] Fahmi M Indonesian higher education: The chronicle, recent development and the new legal entity universities 2007 [http://econpapers.repec.org/paper/unpwpaper/200710.htm] Working Paper in Economics and Development Studies. Department of Economics, Padjadjaran University, Bandung, Indonesia

